# Evaluating the impact of the reconfiguration of gynaecology services at a University Hospital NHS trust in the United Kingdom

**DOI:** 10.1186/1472-6963-14-428

**Published:** 2014-09-24

**Authors:** Teck Choo, Shilpa Deb, Joanne Wilkins, William Atiomo

**Affiliations:** Department of Obstetrics and Gynaecology, Nottingham University Hospitals, Queens Medical Centre, Derby Road, Nottingham, NG7 2UH UK; School of Clinical Sciences, Division of Obstetrics and Gynaecology, University of Nottingham, Nottingham, UK

**Keywords:** Evaluation methodology, Patient satisfaction, Incident reporting, Obstetrics and gynaecology, Patient safety

## Abstract

**Background:**

The project aim was to investigate the impact of reconfiguring gynaecology services on the key performance indicators of a University Hospital NHS Trust in the UK. The reconfiguration involved the centralisation of elective gynaecology on one hospital site and emergency gynaecology on the other.

**Methods:**

Data measuring outcomes of the Trust’s performance indicators (clinical outcomes, patient experience, staff satisfaction, teaching/training, research/development and value for money) were collected. Two time periods, 12 months before and after the reconfiguration in March 2011, were compared for all outcome measures except patient experience. Retrospective data from the hospitals audit department on clinical activity/outcomes and emergency gynaecology patient’s feedback questionnaires were analysed. Staff satisfaction, teaching/training and research/development were measured through an online survey of gynaecology consultants.

**Results:**

Post reconfiguration, the total number of admissions reduced by 6% (6,867 vs 6,446). There was a 14% increase in elective theatre sessions available (902.29 vs 1030.57) and an 84% increase in elective theatre sessions cancelled (44.43 vs 81.71). However, the average number of elective operations performed during each theatre session remained similar (2.63 vs 2.5). There was a significant increase in medical devices related clinical incidents (2 vs 11). With patient experience, there was a significant reduction in patient’s overall length of stay on the emergency gynaecology ward and waiting times for investigations. For staff satisfaction, Consultants were significantly more dissatisfied with workload (3.45 vs 2.85) and standards of care (3.75 vs 2.93). With research and development, consultants remained dissatisfied with time/funding/opportunities for research. No significant impact on undergraduate/postgraduate teaching was found. No financial data on gynaecology was provided for the assessment of value for money.

**Conclusions:**

Reconfiguration of gynaecology services at this Trust may have resulted in a reduction in gynaecological activity and increased cancellation of elective operations but did not significantly reduce the number of elective operations performed. Although consultants expressed increased dissatisfaction with standards of clinical care, clinical incident reports did not significantly increase apart from medical devices incidents. Patient experience of emergency gynaecology services was improved. This manuscript provides a framework for similar exercises evaluating the impact of service redesign in the NHS.

## Background

The UK department of health document “Equity and excellence: Liberating the NHS” published in 2010 [1] and the global economic crisis at the time, outlined a case of need for a viable and efficient UK national healthcare service (NHS) providing high quality care to stakeholders. The key principles were that patients would be at the heart of everything, increasing choice and control, with a relentless focus on clinical outcomes with success measured, not through bureaucratic process targets, but against results that really mattered to patients. A need to empower health professionals was also highlighted with doctors and nurses able to use their professional judgement about what was right for patients and frontline staff would be given more control with healthcare run from the bottom up. Drivers for reconfiguration also included demographic changes, shifting burden of diseases, financial pressures and a need for greater senior medical input (Fulop et al., [2]). A consultation period followed, resulting in launch of a new health and care system from 1 April 2013 to deliver the plans set out in the Health and Social Care Act, with bodies such as NHS England, Public Health England, the NHS Trust Development Authority and Health Education England taking on a range of responsibilities.

With all of these socio-economic drivers [1, 3] came the need to adopt new ways of working and in many instances, a reconfiguration of how services were provided within the NHS. Reconfiguration was described as a deliberately induced change of some significance in the distribution of medical, surgical, diagnostic and ancillary specialties that are available in each hospital or other secondary or tertiary acute care unit in locality, region or health care administrative area (Fulop et al., [2]). It is however very important that mechanisms are put in place to ensure that the impact of service reconfiguration is monitored on an ongoing basis. One bench mark advocated was that any plan for service reconfiguration met four new tests: support from general practitioner (GP) commissioners, strengthened public engagement, consistency with patient choice, and clear evidence for change [4]. The department of health publication, “Equity and excellence: Liberating the NHS” recognises the need for this, with an emphasis on clinically credible and evidence-based outcome measures and not process targets. To date however, there has been very limited published research data objectively evaluating the impact of service reconfiguration on clinical services and staff in the UK NHS. A reconfiguration of the delivery of gynaecology services in Nottingham University Hospitals NHS Trust in 2011 however provided an opportunity to address this need and establish a framework to facilitate similar exercises on a local or national scale.

Nottingham University Hospitals NHS trust was formed in 2006. It is one of the biggest and busiest acute Trusts in England, providing services to over 2.5 million residents of Nottingham and its surrounding communities. At the outset of this project, the Trust had an annual income of £722.5million, 87 wards and about 1,700 beds. The Queens Medical Centre and Nottingham City Hospital are both teaching hospitals and are located 4 miles apart. Both hospitals have a roughly equivalent capacity with respect to the number of in-patient beds.

The vision of the Trust included a determination to be the best acute teaching Trust in the country by 2016. This meant that the Trust’s services and departments would be in the top three compared to peers in the country which would be measured in six areas: clinical outcomes, patient experience, staff satisfaction, teaching and training, research and development and value for money [5].

Gynaecology services in the two main Nottingham Hospitals merged in 2011. The key local drivers for the merger as communicated by the head of service were a need for space on the Queens Medical Centre Campus to accommodate a reconfiguration of other services, cost improvement strategies and to improve patient care. To achieve this plans were made to reduce the number of gynaecology inpatient beds consequent on reduced major inpatient surgery; to increase the number of gynaecological procedures being performed as day cases, leading to an overall reduced length of hospital stay following surgery in gynaecology and finally for consultants to deliver emergency gynaecology services.

Up until March 2011, elective inpatient surgical services were offered on two sites with oncology at the City Hospital Campus and benign and emergency gynaecology services predominantly at the Queens Medical Centre campus. Outpatient services meanwhile were predominantly delivered in the diagnostic and treatment centre located in the Queens Medical Centre Campus managed by an independent healthcare provider. In March 2011, elective inpatient gynaecology services were relocated to the City Hospital Campus with emergency gynaecology services retained at the Queens Medical Centre Campus. This involved the closure of two inpatient gynaecology wards with a new ward renamed the “gynaecology short stay unit” on the Queens Medical Centre campus and the opening of more gynaecology beds on Loxley ward at the City Hospital Campus to accommodate for the move of elective in-patient gynaecology. This reconfiguration led to a reduction in the overall number of inpatient beds. Prior to reconfiguration, emergency gynaecology services were delivered through a nurse led early pregnancy assessment unit (EPAU) and doctor led assessment unit called the general practice admissions unit (GPAU). Patients requiring hospital stay were admitted on to another ward which had capacity for both emergency admissions and elective admissions for elective operations performed at Queens Medical Centre. Following the reconfiguration, all emergency gynaecology services including the EPAU (which continued to be nurse led) GPAU and gynaecology emergency beds were located on a single ward (gynaecology short stay unit) but with a dedicated gynaecology consultant on-call for emergency gynaecology which was not the case before the reconfiguration. There were no concomitant changes to obstetric services and early pregnancy problems such as miscarriages and ectopic pregnancy were managed in the early pregnancy assessment unit within emergency gynaecology services.

These changes had a significant impact on the working patterns of the medical and nursing staffs as staffing rotas were changed to allow 40 hour consultant cover in emergency gynaecology, with consultants on duty and physically present in the hospital when on duty in one week blocks from Monday to Friday from 9-5 pm. The changes were expected to improve the efficiency of gynaecology services, improve quality of patient care and cost effectiveness as demanded by the socio-economic drivers previously outlined.

The aim of this project was to investigate the impact of the reconfiguration of gynaecological services on the key performance indicators of the Trust (clinical outcomes, patient experience, staff satisfaction, teaching and training, research and development and value for money). This was considered important to ensure that standards of patient had not been compromised, staff retention and morale had been maintained, the changes had not negatively impacted on education and research and that any changes undertaken had ensured value for money.

## Methods

### Study design

The planned project was presented at the local gynaecology consultants meeting on the 16^th^ of December 2011. This was essentially a natural experiment. A decision had been undertaken to make some changes to the delivery of the service which provided the opportunity for a before and after observational study. Formal research ethics approval and informed written consent was not thought to be required as the project was a service evaluation. Data was collected to measure outcome measures categorised in accordance with the Trust’s six performance indicators. The main categories were clinical outcomes, patient experience, staff satisfaction, teaching and training, research and development and value for money. Data was retrospectively collected from the hospitals information technology (IT), coding and audit departments on clinical outcomes. For patient experience, a patient feedback questionnaire on patient’s experience of emergency gynaecology services at the Trust, designed by a gynaecology consultant (SD) appointed as lead for emergency gynaecology services in July 2010 were retrospectively analysed. The impact of the changes on staff satisfaction, teaching and training and research and development was measured using a prospectively administered questionnaire (see below). Data to evaluate the impact of the reconfiguration of gynaecology services on value for money was not received from the finance department, we were told because gynaecology financial data was not separately ring fenced from obstetrics. Two time periods were compared for all outcome measures except for patient experience; the 12 months leading up to the reconfiguration in March 2011 (March 2010 to February 2011) and the 12 months after (March 2011 to February 2012). For patient experience, questionnaire collection was commenced in October 2010 to January 2011 (pre re-configuration) and post reconfiguration questionnaire collection was performed from July 2011 to May 2012. The patient questionnaire was only collected from emergency gynaecology patients. The methods of evaluation for the trust key performance indicators are summarized in Table 1.

**Table 1:** **Summary of method of evaluation for the trust key performance indicators**

Trust key performance indicator	Method of evaluation
Clinical outcomes	All reported clinical incidents, Clinical throughput/activity data obtained from the Trust’s patient safety incidents healthcare software on the clinical incidents reported (Datix).
Patient experience	Patient feedback questionnaire on patient’s experience of emergency gynaecology services
Staff satisfaction	The Measure of Job Satisfaction (MJS) questionnaire sent to Gynaecology Consultants in the department
Teaching and training and	Two questions asking about the time and opportunity to provide teaching and training sessions for undergraduates and postgraduates, included in the MJS questionnaire.
Research and development	Three questions asking about time to carry out research, funding for research and opportunities for research, included in the MJS questionnaire.

### Measuring clinical outcomes

Clinical outcomes were measured based on the clinical incidents reported and the clinical throughput/activity data which were collected from the IT, coding and audit department. Clinical throughput/activity data was measured by comparing the total number of admissions, operations and elective theatre utilisation in gynaecology in the 12 months leading up to the reconfiguration with the 12 months after reconfiguration. Clinical incidents were measured using data obtained from Datix, the trust’s patient safety incidents healthcare software on the clinical incidents reported. The incidents reported were categorised into incidents related to treatment and procedure, documentation and health records, patient injury/accidents, patient care, medication, access, admission, transfer and discharge and blood and blood products, diagnosis scans and tests, equipment /medical devices (clinical) and communication. Actual clinical incidents under each category were then evaluated. Only data from patients treated at the Nottingham University Hospitals NHS trust were included with data from the nearby NHS diagnostic and treatment centre excluded from the analysis as it was not affected by the reconfiguration. Gynaecology-oncology patients were also included in the data.

### Patient experience

The key variables measured by the questionnaire included; reasons for attendance, communication, waiting times, environment and overall experience of the service measured based on free text responses to two questions (“What do you think we did well today?” and “What do you think we could have done better?”).

### Staff satisfaction

For staff satisfaction, a literature search was performed to identify a questionnaire to measure job satisfaction. The Measure of Job Satisfaction (MJS) questionnaire was considered the most relevant validated questionnaire used for healthcare workers [6, 7] as it was specifically designed to monitor the morale of healthcare staff following changes in legislation and the delivery of health and social care in the U.K and it was sensitive to differences in satisfaction over time. The hospital consultant stress and job satisfaction questionnaire [8] was also considered but not used as it had not been as extensively used as the MJS and mainly focused on mental health.

The MJS questionnaire used was divided into 7 categories, which were personal satisfaction, satisfaction with workload, satisfaction with professional support, satisfaction with training, satisfaction with pay, satisfaction with prospects and satisfaction with standards of care. The questionnaire was anonymously sent via an online survey to 13 Gynaecology Consultants in the department excluding the 4 gynaecology oncologists as their elective work remained primarily on one hospital campus before and after the reconfiguration. The survey measured job satisfaction in the twelve months leading up to the reconfiguration in March 2011 (March 2010 to February 2011) and the twelve months after (March 2011 to February 2012). Of the 13 consultants surveyed, 5 consultants did only gynaecology and the rest had a workload which combined obstetrics and gynaecology. Eight consultants worked primarily on the Queens Medical Centre campus site and five at the City Hospital site.

### Teaching and training and research and development

To evaluate the impact of the reconfiguration on teaching and training, two questions added to the online survey asking about the time and opportunity to provide teaching and training sessions for undergraduates and postgraduates were included in the MJS questionnaire. For research and development there were three questions included into the online survey. Consultants were asked about their opinion regarding time to carry out research, funding for research and opportunities for research.

### Data analysis and statistics

The proportion of each group of clinical incidents was calculated, with the denominator being either total operations or total admissions depending on the incident. The three incidents where total operations were used as the denominator were operations cancelled, unexpected returns to theatre and incorrect or inappropriate procedure/operation/side of body. All the other incidents had their percentages calculated with their denominator as total admissions. The paired T-Test was the most appropriate test to use to compare two groups as advised by our statistician. Using the statistical software SPSS, a paired T-Test was performed to compare if there were any significant difference between the incidents before and after the reconfiguration of Gynaecological services.

The MJS questionnaire comprised of 7 subscales which were combined to give a measure of ‘Overall Job Satisfaction’. There were 43 items on the questionnaire all of which were scored 1 to 5 as follows: very dissatisfied =1, Dissatisfied = 2, neither satisfied nor dissatisfied = 3, Satisfied = 4, Very satisfied = 5. The first 43 items formed 7 subscales of job satisfaction. Item mean scores were calculated for each subscale by dividing the sum of item scores by the number of items comprising that scale. For example, the ‘Satisfaction with Standards’ scale consisted of 6 items. The item mean score was therefore the sum of all items divided by 6. Similarly ‘Overall Job Satisfaction’ was the sum of the first 43 items divided by 43. The last question, item 44, gave an indication of global satisfaction.

Results from all questionnaires completed and returned were summarised on a spreadsheet and the overall/group mean of the item mean scores of the 7 subscales on the MJS and the ‘Overall Job Satisfaction’ calculated. A paired T-test was performed with the SPSS software to find out if there were any statistically significant differences in job satisfaction, on comparing the twelve months leading up to the reconfiguration in March 2011 (March 2010 to February 2011) with the twelve months after (March 2011 to February 2012). Similarly, to evaluate teaching and training and research and development, group mean satisfaction scores for the additional questions assessing teaching and research were compared using the paired T-test to find out if there were any statistically significant differences on comparing the twelve months leading up to the reconfiguration in March 2011 (March 2010 to February 2011) with the twelve months after (March 2011 to February 2012).

For patient experience quantitative data (proportions) on waiting times on the emergency gynaecology unit and overall experience of the service were compared in the period pre-reconfiguration captured by the questionnaire (October 2010 to January 2011) with the period post reconfiguration (July 2011 to May 2012 ). A p value of less than 0.05 was accepted as statistically significant for all data analysis.

## Results and discussion

### Results

#### Clinical outcomes

1. Clinical activity

Clinical activity is shown in Table 2. There was a 6% reduction (6867 to 6446) in the total number of gynaecological admissions to the Trust in the year following the reconfiguration compared to the year before with a 17% reduction (1055 to 880) in day-case admissions, a 9% reduction (2308 to 2108) in inpatient admissions and a 1% reduction (3504 to 3458) in emergency admissions across both hospital sites where gynaecology services are delivered. There was also a 4% reduction (2961 to 2856) in the total number of operations performed. Although the total number of elective operations increased by 3%, (2239 to 2298) there was a 22% reduction in emergency gynaecology surgery across both hospital sites where gynaecology services are delivered (668 to 547). Emergency gynaecological surgery reduced by 24% (667 to 514) at the Queens Medical Centre Site and increased by 50% (22 to 33) at the City Hospital site (most likely a reflection of returns to theatre following elective gynaecological surgery at the City Hospital site).

**Table 2:** **Clinical activity**

Clinical throughput/activity data in gynaecology before and after the reconfiguration of services in March 2011			
	March 2010 to February 2011	March 2011 to February 2012	Percentage change
ADMISSIONS			
Emergency	3,504	3,458	-1%
Inpatient	2,308	2,108	-9%
Day case	1,055	880	-17%
Grand total	6,867	6,446	-6%
TOTAL OPERATIONS			
ELECTIVE; - CITY	1,337	2,281	71%
QUEENS	902	17	-98%
ELECTIVE Total	2,239	2,298	3%
EMERGENCY;- CITY	22	33	50%
QUEENS	677	514	-24%
EMERGENCY Total	699	547	-22%
URGENT CITY	1		-100%
QUEENS	22	11	-50%
URGENT Total	23	11	-52%
Grand Total	2,961	2,856	-4%
THEATRE UTILISATION			
Planned nominal sessions available	902.29	1030.57	14%
Number of nominal sessions used	849.57	918.43	8%
Nominal sessions cancelled	44.43	81.71	84%
Cancelled <3wks			
Nominal sessions not canc not used	8.29	30.43	267%
Nominal sessions reinstated	22.86	59.14	159%
Nom. sess. reinstated from diff spec	8.14	0.00	-100%
Planned time (Hrs)	3158.00	3607.00	14%
Actual time (Hrs)	2973.50	3214.50	8%
Cancelled time (Hrs)	155.50	286.00	84%
Not canc not used (Hrs)	29.00	106.50	267%
Nominal sessions reinstated (Hrs)	80.00	207.00	159%
Session utilisation	94.16%	89.12%	-5%

With regards to theatre utilisation (Table 2), pre-reconfiguration, 902.29 sessions were available to gynaecology of which 849.57 (94.16%) were actually used compared with 1030.57 in the year post reconfiguration of which 918.43 (89.12%) were actually used. 44.43 theatre sessions were cancelled pre-reconfiguration compared with 81.71 post-reconfiguration (84% increase). The average number of elective operations performed during each theatre session however remained about the same (2.63 pre reconfiguration and 2.5 post reconfiguration).

2. Clinical incidents

Clinical incidents are shown in Table 3. There was a significant increase in the incidents reported in the category of Equipment / Medical Devices (Clinical). Subdividing this category, it appears that the significant increase was in the area of unavailability of equipment (patient safety). There were no statistical differences in the other nine categories of incidents reported.

**Table 3:** **Clinical incidents**

	Categories of incidents reported		Pre-reconfiguration	Post- reconfiguration	P- value
	1 Treatment and procedure		23 / 6844	18 / 6428	0.562
	2 Documentation and health records		19 / 6848	18 / 6428	0.978
	3 Patient care		11 / 6856	17 / 6429	0.192
	4 Medication		12 / 6855	16 / 6430	0.355
	5 Access, admission, transfer and discharge		18 / 6849	9 / 6437	0.116
	6 Blood and blood products		17 / 6850	7 / 6439	0.059
	7 Diagnosis, scans and tests		4 / 6863	11 / 6435	0.053
	8 Equipment / medical devices (clinical)		2 / 6865	11 / 6435	0.009
		Unavailability of equipment (Patient safety)	1 / 6866	6 / 6440	0.048
		Equipment fault or damage (Patient safety)	1 / 6866	4 / 6442	0.158
		User error (Patient safety)	0 / 6867	1 / 6445	0.302
	9 Communication		4 / 6863	8 / 6438	0.206
	10 Infection control		2 / 6865	1 / 6445	0.601

*Numbers in columns pre-reconfiguration and post reconfiguration = incidents reported / no incident reports.*

### Patient experience

Seventy-one questionnaires in total were available for analysis in the period before the reconfiguration and 208 post-reconfiguration. The difference in total numbers analysed for each question was due to the fact that not each patient attended for the same thing and therefore some questions were not applicable for them to answer and were therefore left blank. There was a no significant difference in the time spent on the ward before patients were seen by a doctor or nurse. However, following the reconfiguration, there was a significant reduction in the length of time patients waited before having an ultrasound scan (post reconfiguration, 65% of patients had their ultrasound scans within 20 minutes compared with 39.5%% before; p = 0.0054), 54% of patients had their blood test result within 20 minutes compared with 0% before; p = 0) and the overall length of stay on the ward (Table 4) was reduced with 61% of patients staying on the ward for less than one hour compared with 22% pre-reconfiguration; p = 0). With regards to the overall experience of the service, 47% of free comments suggested no improvements were required pre-reconfiguration compared to 44% post-reconfiguration. There was however a 22% increase in comments suggesting that waiting times could be improved and an 11% and 13% decrease in comments suggesting that the environment and communication could be improved post-reconfiguration. Paradoxically, in response to the question “what did we do well today?”, there was a 13.5% increase in comments relating to waiting times and a 12.5% reduction in comments relating to communication (Table 5).

**Table 4:** **(Patient experience) Waiting times (Emergency gynaecology wards)**

			Pre-reconfiguration number,n (%)	Post-reconfiguration number n, (%)	p value
Time waiting on the ward before patient seen by a doctor or nurse	Wait time	5-20 mins	48 (74)	136 (65)	0.16
		25-40 mins	8 (12)	32 (16)	0.404
		45-60 mins	7 (11)	29 (14)	0.514
		>60mins	2 (3)	11 (5)	0.445
	Total number of responses		65	208	
How long did you wait for an ultrasound (if applicable)	Wait for Ultrasound	0-20 mins	15 (39.5)	95 (65)	0.0045
		20-40 mins	15 (39.5)	30 (22)	0.045
		40-60 mins	6 (16)	13 (9)	0.2802
		>60 mins	2 (5)	7 (5)	1
	Total number of responses		38	145	
How long did you wait for a blood test (if applicable)	Wait for blood test	0 - 20 mins	4 (36.4)	19 (54.3)	0.2934
		30 - 60 mins	4 (36.4)	12 (34.3)	0.9214
		> 60 mins	3 (27.2)	4 (11.4)	0.3003
	Total number of responses		11	35	
How long did you wait for the results of your blood test (if applicable)	Wait for results of blood test	0 - 25 mins	0 (0)	9 (54)	0
		60 - >60 mins	2 (100)	8 (47)	0
	Total number of responses		2	17	
How long did you stay on the ward	Length stayed on ward	<1 hr	11( 22)	44 (61)	0
		1 - 4 hrs	18 (36)	20 (28)	0.35
		>4 hrs	21 (42)	8 (11)	0.0001
	Total number of responses		50	72

**Table 5:** **(Patient experience) Free text comments**

Free text comments			
	Pre-reconfiguration	Post-reconfiguration	
Q 15 What do you think we did well today?	Number of responses (%)	Number of responses (%)	Difference (%)
Communication	20 (34)	18 (21.5)	-12.5
Staff	26 (44)	36 (43)	-1.0
Waiting time	5 (8)	18 (21.5)	13.5
Environment	4 (7)	2 (2)	-5.0
Overall good	4 (7)	10 (12)	5.0
Total number of comments	59	84	
**Q 16 What do you think we could have done better?**			
No improvements	16 (47)	19 (44)	-3.0
Waiting time	5 (15)	16 (37)	22.0
Staff	0 (0)	2 (5)	5.0
Environment	8 (23)	5 (12)	-11.0
Communication	5 (15)	1 (2)	-13.0
Total number of comments	34	43	

### Staff satisfaction

Consultant satisfaction questionnaire: 10 out of 13 (77%) consultants replied. There was a significant decrease in the mean score for consultant’s satisfaction with workload from 3.45 to 2.85 (P = 0.000) (Table 6). There was also a significant decrease in the average score for satisfaction with standards of care from 3.75 to 2.93 (P = 0.000). Both these results showed a change from “neither satisfied nor dissatisfied” to being “dissatisfied” after reconfiguration. Two areas showed significant change but satisfaction remained in the same categories. Satisfaction with professional support changed from a mean of 3.66 to 3.15 (P = 0.002). Similarly, there was a significant change in satisfaction with prospects (P = 0.015) from a mean of 3.65 to 3.43. Both scores remained in the same category of being “neither satisfied nor dissatisfied”. The average overall satisfaction among consultants showed a significant reduction from 3.63 to 3.26 (P = 0.000) after reconfiguration. However, this score remained in the same category of being “neither satisfied nor dissatisfied”.

**Table 6:** **Measurement of Job Satisfaction (MJS)**

		Mean score	Satisfaction score (Mean/10)	N (Number of questions)	Std. Deviation	Sig. (2-tailed)
Personal satisfaction	Pre- reconfiguration	38.17	3.81	6	3.43	0.633
	Post- reconfiguration	37.33	3.73	6	4.761	
Satisfaction with workload	Pre- reconfiguration	34.50	3.45	8	2.07	0.00
	Post- reconfiguration	28.50	2.85	8	3.464	
Satisfaction with professional support	Pre- reconfiguration	36.62	3.66	8	1.50594	0.002
	Post- reconfiguration	31.50	3.15	8	2.9761	
Satisfaction with training	Pre- reconfiguration	34.60	3.46	5	3.20936	0.089
	Post- reconfiguration	32.60	3.26	5	3.04959	
Satisfaction with pay	Pre- reconfiguration	37.25	3.72	4	2.06155	0.174
	Post- reconfiguration	34.50	3.45	4	2.38048	
Satisfaction with prospects	Pre- reconfiguration	36.50	3.65	6	2.25832	0.015
	Post- reconfiguration	34.33	3.43	6	2.58199	
Satisfaction with standards of care	Pre- reconfiguration	37.50	3.75	6	1.22474	0.00
	Post- reconfiguration	29.33	2.93	6	2.42212	
Overall satisfaction	Pre- reconfiguration	36.37	3.63	43	2.517	0.00
	Post- reconfiguration	32.26	3.22	43	4.215	

*(Very dissatisfied =1, Dissatisfied = 2, neither satisfied nor dissatisfied = 3, Satisfied = 4, Very satisfied = 5).*

### Teaching and training and research and development

The questions asked in the questionnaire to assess the impact on research were time to carry out research, funding for research and opportunities for research. The results showed a significant change in a mean score of 2.86 to 2.70 (P = 0.038) (Table 7), this however remained in the same category of being “dissatisfied”. The questions asked in the questionnaire to assess the impact on teaching were time and opportunities to provide undergraduate teaching sessions and the same for postgraduates. The results showed a similar opinion of being “neither satisfied nor dissatisfied” (mean of 3.55 to 3.45, p = 0.500).

**Table 7:** **Research and teaching**

		Mean	Satisfaction score (Mean/10)	N (number of questions)	Std. Deviation	Sig. (2-tailed)
Research	Pre- reconfiguration	28.67	2.86	3	1.528	0.038
	Post- reconfiguration	27.00	2.70	3	1.000	
Teaching	Pre- reconfiguration	35.50	3.55	2	0.707	0.500
	Post- reconfiguration	34.50	3.45	2	0.707	

*(Very dissatisfied =1, Dissatisfied = 2, neither satisfied nor dissatisfied = 3, Satisfied = 4, Very satisfied = 5).*

### Discussion

This is the first study as far as we know which has objectively measured the impact of service reconfiguration in gynaecology on a range of measures. This study is limited to a single department in a single region, the findings may not be generalizable to other settings. Patient safety did not appear to have been significantly compromised as there were no statistical differences in 9 out of the 10 categories under which clinical incidents are reported. There was however a significant increase in the incidents reported in the category of “equipment / medical devices” mainly due to the unavailability of equipment (increased from one incident to six incidents reported) and equipment fault or damage (increased from one incident to four reported). Following the reconfiguration, there was a significant reduction in the length of time patients waited before having an ultrasound scan, and blood test result and the overall length of stay on the gynaecology emergency ward. This difference mainly could be due to the presence of dedicated consultant on-call for emergency gynaecology providing senior input early in the patient care pathway, as described in the report by the Academy of Royal Colleges 2012 [9].

The results also showed a 6% reduction in total gynaecological admissions in the one year following the reconfiguration of services compared to the year before the reconfiguration, with the most noticeable drop in the number of day-case admissions (17%) possibly because more day-case operations were being performed in the Nottingham Diagnostic and Treatment centre. There was also a 4% reduction in the total number of operations performed (emergency and elective) which was mainly accounted for by the significant decrease in the number of emergency operations (22%) in spite of a 3% increase in the number of elective operations. This again could be attributed to an early senior input in management of emergencies [9]. To accommodate for the dedicated consultant on call cover, their elective theatre sessions had to be cancelled thereby contributing partly to the 84% increase in the number of nominal/elective theatre sessions cancelled. Changes in clinical activity data could also have been due to random fluctuations and chance. Factors external to the hospital, such as changes in commissioning structures, could also have played a role.

With regards to staff satisfaction, staff surveyed (consultants) were more dissatisfied with the standards of clinical care provided and workload, following the reconfiguration of services, but remained neutral about their overall job satisfaction, prospects and professional support. Consultants were already dissatisfied with the time, funding and opportunities to carry out research prior to reconfiguration of gynaecological services and remained dissatisfied in the year after. The changes may have resulted in less time available for consultants to seek and apply for funding for research grants because of changes to their working hours/job plans, however there were no significant differences from the survey analysis. There was no significant impact on undergraduate or postgraduate teaching.

We acknowledge that a limitation of the study was the absence of a control group to determine whether the magnitude of the change would have occurred anyway even if the changes hadn’t been put in place. We are however unable to locate a similar Trust where exactly the same changes were effected in the same period to be able to make any valid comparisons. It is difficult to speculate on how these findings compare with previous research as there is limited research data measuring the impact of service reconfiguration on organisational outcomes. Although there were editorials, letters, opinion pieces and studies evaluating the implementation process [10–18] only three studies [15–17] were identified where the impact of a reconfiguration of clinical services on any pre-defined outcomes was measured and no published study was identified measuring the impact of reconfiguration of gynaecology services. One ongoing study was identified measuring the impact of the reorganisation of stroke services in London and Greater Manchester on quality of care and patient outcomes, but the results are not yet available [18]. In the first published study we identified, [15] neuro-oncology service reconfiguration in accordance with the National Institute for Health and Care Excellence (NICE) guidance resulted in enhanced clinical care for patients with glioblastoma multiform when data from the Anglian Cancer Network was analysed. In the second study [16], there was no increase in mortality due to the centralisation of acute surgical services in the Mid-West of the republic of Ireland in the year before compared with the year after surgical service reconfiguration. In the third study [17], the effects of externally led redesign over 6 months within two hospitals, comprising ward-based innovations led by consultancy-led standardized processes, and internally led redesign over 25 months in one hospital which implemented medical assessment and planning unit, 23 h elective surgical ward and new bed management processes were compared in a retrospective, before–after study involving five tertiary hospitals in Queensland, Australia. The results showed that in all the hospitals there was a decline in waits for elective surgery waits with oscillations in data indicating the existence of confounding variables other than redesign. The lack of increase in mortality in the Irish study [16] is consistent with our results where we found no significant increases in 90% of the categories under which clinical incidents are reported which was reassuring. It is however worth noting that the findings of these are not necessarily applicable a) to the English NHS (as they relate to changes overseas), because of differences in health systems and b) to gynaecology services. Although in many reconfigurations the drivers are similar, each set of proposals will have its own unique context.

It is difficult to attribute the reduction in total gynaecological admissions and operations following reconfiguration primarily to the reconfiguration of services as there were also parallel changes to the working pattern of consultants in gynaecology, staffing changes and external bed pressures following the reconfiguration which could have acted as potential confounding variables. For example, the introduction of “hot weeks” in gynaecology emergency (one week blocks where consultant gynaecologists are on call for emergency gynaecology from 8 am to 5 pm from Monday to Friday, with all their elective work cancelled) in parallel with the reconfiguration, improved the availability senior medical staff. This could have explained the significant reduction in the emergency admissions, emergency surgery, waiting times for investigation and diagnosis and the overall length of stay on the gynaecology emergency ward found on the questionnaires measuring patient satisfaction. Although staff satisfaction questionnaires showed increased dissatisfaction in the standards of clinical care provided and workload, this was not reflected in the measures of patient safety. It could however be argued that these views may have been attributed to the change in working patterns, increased pressures on the waiting times for elective procedures, financial remuneration and the change in working environment for both the staff and patients especially as two key events occurred at the same time. Ward moves which were disruptive probably due to the cross site travel and “hot weeks” which changed the consultant working pattern, but improved patient experience, waiting times and reduced emergency surgery.

The strengths of this study were in its novelty and the objective way in which outcomes were selected and measured. It would have been ideal to incorporate individual patient demographic and other data to eliminate the potential of bias from a different type of patient accessing the serviced after reconfiguration. However we did not have this data and it would only have been relevant for a component of one out of the six key outcomes of interest (clinical outcomes). It would have been ideal for the questionnaires evaluating patient experience to have been obtained from two similar service set-ups and time frames to enable a more objective comparison especially as we were unable to clarify from the questionnaires which specific patient experience questionnaires were received primarily from early pregnancy assessment unit (EPAU) and which ones were emergency gynaecology admissions. It would also have been ideal to evaluate the impact of the reconfiguration on value for money with the inclusion of financial data. The 6% reduction in gynaecological admissions would suggest that there may also have been reduction in income however we did not have data on expenses to enable an assessment on margins (profit/losses) as the financial data includes both the obstetrics and gynaecology department. On the other hand, this could also be interpreted as a favourable outcome as this could have been due to better patient care pathways and guidelines between primary care, the emergency department and secondary care and dedicated senior consultant level cover. It is however important for future studies that an evaluation (including financial) strategy is embedded in reconfiguration plans at the outset to enable accurate measurement of the financial impact of any proposed changes.

The consultant satisfaction data were collected at a single point in time, several months after the reconfiguration. This may have introduced an element of recall bias, particularly if the change process had been challenging. There may also have been other causes for dissatisfaction, which were not captured by this quantitative survey approach. The data were also only collected for 12 months after the reconfiguration. It may take time for changes to ‘bed in’ and staff to get used to new ways of working, and hence the activity data may stabilise in time.

## Conclusions

In conclusion, this study set out to investigate the impact of a reconfiguration of gynaecological services on the key performance indicators of a University NHS Trust in the United Kingdom. Patient safety did not appear to have been significantly compromised following the reconfiguration. Patients presenting as an emergency experienced a reduction in the length of time they had to wait for key investigations and results and an overall reduction in their length of stay on the gynaecology emergency ward following reconfiguration. There was however a reduction in total gynaecological admissions to the Trust probably due to the ability to perform more complex cases in the day case setting provided by the Nottingham Treatment centre in the year following the reconfiguration. The 84% increase in the number of elective theatre sessions cancelled may have been due to the dedicated consultant cover for emergency gynaecology and possibly the winter pressures on the shrinking overall bed capacity. Although the Consultants expressed overall satisfaction, they were dissatisfied with the standards of clinical care provided, workload, and opportunities for research. There was however no significant impact on undergraduate or postgraduate teaching.

This manuscript provides a framework for similar exercises evaluating the impact of service redesign on a local, regional or national scale especially in the rapidly changing complex climate in which high quality and safe clinical services have to be delivered in the modern NHS.

## Abbreviations

NHS: National Health Service
GP: General practitioner
EPAU: Early pregnancy assessment unit
GPAU: General practice admissions unit
IT: Information technology
MJS: Measure of job satisfaction questionnaire
NICE: National Institute for Health and Care Excellence
TC: Teck Choo (author)
SD: Shilpa Deb (author)
JW: Joanne Wilkins (author)
WA: William Atiomo (author).
